# Mineral trioxide aggregate immersed in sodium hypochlorite reduce the osteoblastic differentiation of human periodontal ligament stem cells

**DOI:** 10.1038/s41598-021-01545-3

**Published:** 2021-11-11

**Authors:** Kozue Yamashita, Atsushi Tomokiyo, Taiga Ono, Keita Ipposhi, M. Anas Alhasan, Akira Tsuchiya, Sayuri Hamano, Hideki Sugii, Shinichiro Yoshida, Tomohiro Itoyama, Hidefumi Maeda

**Affiliations:** 1 Department of Endodontology and Operative Dentistry, Faculty of Dental Science, Kyushu University, 3-1-1 Maidashi, Higashi-ku, Fukuoka-shi, Fukuoka 812-8582 Japan; 2 Department of Endodontology, Kyushu University Hospital, 3-1-1 Maidashi, Higashi-ku, Fukuoka-shi, Fukuoka 812-8582 Japan; 3 OBT Research Center Faculty of Dental Science, Kyushu University, 3-1-1 Maidashi, Higashi-ku, Fukuoka-shi, Fukuoka 812-8582 Japan; 4 Department of Biomaterials, Faculty of Dental Science, Kyushu University, 3-1-1 Maidashi, Higashi-ku, Fukuoka-shi, Fukuoka 812-8582 Japan

**Keywords:** Calcium-based cement, Dental biomaterials, Mineral trioxide aggregate, Endodontics

## Abstract

White mineral trioxide aggregate (WMTA) is a root canal treatment material, which is known to exhibit a dark brown color when in contact with sodium hypochlorite solution (NaOCl). This study aimed to investigate the effects of NaOCl on the surface properties of WMTA discs and WMTA-induced osteoblastic differentiation of periodontal ligament stem cells (PDLSCs). Mixed WMTA (ProRoot MTA) was filled into the molds to form WMTA discs. These discs were immersed in distilled water (D-WMTA) or 5% NaOCl (Na-WMTA). Their surface structures and Ca^2+^ release level was investigated. Moreover, they were cultured with a clonal human PDLSC line (line 1–17 cells). The main crystal structures of Na-WMTA were identical to the structures of D-WMTA. Globular aggregates with polygonal and needle-like crystals were found on D-WMTA and Na-WMTA, which included Ca, Si, Al, C and O. However, many amorphous structures were also identified on Na-WMTA. These structures consisted of Na and Cl, but did not include Ca. NaOCl immersion also reduced Ca^2+^ release level from whole WMTA discs. Line 1–17 cells cultured with D-WMTA formed many mineralized nodules and exhibited high expression levels of osteoblast-related genes. However, cells incubated with Na-WMTA generated a small number of nodules and showed low expression levels of osteoblast-related genes. These results indicated that NaOCl reduced Ca^2+^ release from WMTA by generating amorphous structures and changing its elemental distribution. NaOCl may also partially abolish the ability of WMTA to stimulate osteoblastic differentiation of PDLSCs.

## Introduction

A root perforation represents a mechanical or pathological communication between the root canal system and the periodontium or oral cavity^1^. Unsealed root perforations impair the health of periapical tissues because they are exposed to microorganisms and other contaminants, which can result in microbial infection in the root canal system. A previous report demonstrated that the duration of perforation exposure to contamination is an important prognostic factor for teeth with root perforations^2^. Therefore, the success of root canal treatment for teeth with root perforations requires immediate sealing to eliminate the risk of bacterial infection. Accordingly, root canal treatment is often performed after perforation repair.

The feasibility of root perforation sealing is also crucial for the prognosis of a perforated tooth^2^. Mineral trioxide aggregate (MTA) has been regarded as an ideal dental material for perforation repair because of its sealing ability, biocompatibility, and antibacterial potential^3^. Additionally, MTA induces the formation of hard tissues in root perforation areas^4^. The first commercially available MTA was a gray-colored MTA, which was likely to darken overlying tissues. However, a white-colored MTA (WMTA) was developed in 2002. WMTA differs from gray-colored MTA in terms of its substantial reduction in the proportion of the tetra calcium aluminoferrite component^5^; therefore, it could address esthetic concerns associated with the use of gray-colored MTA and has been commonly used worldwide.

The principal aim of root canal treatment is to eliminate existing bacteria in the root canal system. Sodium hypochlorite solution (NaOCl) has strong antimicrobial effects against bacteria (including bacteria in biofilms), fungi, and viruses; it can dissolve organic compounds including pulpal tissues^6^. Therefore, NaOCl is regarded as the preferred root canal irrigant. However, several reports have described its effects on WMTA; contact between WMTA and NaOCl results in dark brown discoloration of WMTA^7^, as well as reduced formation of calcium hydroxide (Ca [OH]_2_)^8^.

Somatic stem cells play crucial roles in tissue homeostasis and regeneration. Among components of the periodontium, periodontal ligament (PDL) has been reported to contain PDL stem cells (PDLSCs) that possess self-renewal capacity and potential for differentiation into osteoblasts, cementoblasts, and adipocytes^9^. Additionally, WMTA facilitated the differentiation of PDLSCs into mature cell types (e.g., osteoblasts and cementoblasts)^10^, suggesting that WMTA filled to the perforated sites induced the formation of hard tissues by promoting osteoblastic differentiation in PDLSCs.

We established a multipotent clonal PDLSC line, line 1–17 cells, in 2008^11^. Briefly, human PDL cells isolated from the health premolar of a 20-year-old female were immortalized by transfection of the cells with Simian virus 40 large T-antigen and human telomerase reverse transcriptase^12^. Line 1–17 cells were isolated from them by a limiting dilution. This line showed typical spindle-shaped fibroblastic morphology and expressed the mesenchymal stem cells (MSC) -related markers STRO-1, CD13, CD29, CD44, CD71, CD90, CD105, CD146, and CD166^13^. They also expressed the embryonic stem cell (ESC) -related pluripotency genes *OCT4* and *Nanog* and neural crest (NC) -related marker genes *p75NTR*, *SLAG*, *SOX10*, *NESTIN*, and *CD49D.* Moreover, they could differentiate not only into mesenchymal cells like osteoblasts, adipocytes, and chondrocytes but also into ectoderm cells like neural cells. Intriguingly, line 1–17 cells strongly expressed the PDL-related molecules periostin and scleraxis as well as MSC-. ESC-, and NC-related markers and revealed the potential to generate bone/cementum-like tissues following the subcutaneous transplantation of both lines into the dorsal side of immunodeficient mice^14^.

To our knowledge, no study has been published regarding the effects of NaOCl-exposed WMTA on osteoblastic differentiation in PDLSCs. Therefore, this study aimed to characterize the structure of WMTA treated with NaOCl and to evaluate its ability to induce differentiation in a clonal human PDLSC line.

## Materials and methods

### WMTA disc formation

WMTA powder (ProRoot MTA; Dentsply Sirona, Charlotte, NC, USA) was mixed with distilled water (DW) at a ratio of 0.33. It was cured in a silicon mold (5 mm diameter and 2 mm height) for 24 h at 37 °C and 100% humidity to form WMTA discs. These discs were immersed in DW (D-WMTA) or 5% sodium hypochlorite solution (NaOCl; WAKO, Osaka, Japan) (Na-WMTA) for 24 h.

### X-ray diffraction (XRD) analysis

Crystalline structures of WMTA discs without immersion (Cont-WMTA), D-WMTA, and Na-WMTA were investigated by XRD (D8-Advance A25, Bruker, Karlsruhe, Germany) using CuKα radiation operated at 40 kV and 40 mA. Theoretical diffraction patterns of bismite (Bi_2_O_3_, PDF#00-002-0498), calcite (CaCO_3_, PDF#00-002-0629), calcium silicate (Ca_2_SiO_4_, PDF#00-002-0843), and calcium hydroxide (Ca [OH]_2_, PDF#01-070-5492) were calculated by using values reported in the literature for the corresponding crystal structures.

### Scanning electron microscope (SEM) and energy dispersive X-ray spectrometer (EDX) analysis

Morphological evaluations of Cont-WMTA, D-WMTA, and Na-WMTA were performed by SEM (S-3400N; Hitachi High-Technologies Co., Tokyo, Japan) using an accelerating voltage of 15 kV, following gold/palladium coating. Elemental compositions of these discs were analyzed by EDX (Genesis XM4; EDAX, Mahwah, NJ, USA). Elemental maps were plotted for O, C, Ca, Na, Si, Bi, Cl, and Al.

### Ca^2+^ release and pH level evaluation

The wells of 48-well culture plates (Becton Dickinson Labware, Lincoln Park, NJ, USA) were filled with 500 µl of α-MEM (Gibco-BRL, Grand Island, NY, USA) supplemented with 50 µg/ml streptomycin and 50 U/ml penicillin (Penicillin–Streptomycin Solution [×100], Wako, Osaka, Japan) containing 10% fetal bovine serum (Biosera; Nuaillé, France) (CM). Then, D-WMTA or Na-WMTA were placed on the bottoms of wells (one disc per well). Wells filled with CM without WMTA discs (No WMTA) were prepared as controls. All plates were maintained at 37 °C in a humidified atmosphere of 5% CO_2_ and 95% air. Media was collected on days 1, 7, 14, and 28 (n = 3 discs per time point). The amount of Ca^2+^ release was assessed with a QuantiChrom Calcium Assay Kit (Bio Assay Systems, Hayward, CA, USA), then measured in accordance with the manufacturer’s instructions using a microplate reader at an absorbance of 590 nm. The pH level was measured by Twin pH Meter II LQUA twin (Horiba Advanced Techno, Kyoto, Japan).

### Culturing line 1–17 cells with WMTA discs

A multipotent clonal human PDLSC line, line 1–17 cells, was used in this study. We established this line in 2008^11^. Briefly, human PDL cells (HPDLCs) were isolated from the health premolar of a 20-year-old female who visited the Dental Hospital of Kyushu University for extractions. Informed consent was obtained from the patient prior to the study. Then, HPDLCs were immortalized by transfection of the cells with Simian virus 40 large T-antigen and human telomerase reverse transcriptase^12^. Line 1–17 was isolated from them by a limiting dilution. This line was cultured in CM at 37 °C in a humidified atmosphere of 5% CO_2_ and 95% air. All procedures were carried out following the rules of the Declaration of Helsinki and in accordance with the requirements of the Kyushu University Certified Institutional Review Board for Clinical Trials. The study was approved by Kyushu University Certified Institutional Review Board for Clinical Trials (approval number: 2–115). Following incubation of D-WMTA and Na-WMTA in 500 µl of α-MEM for 24 h, the discs were washed with phosphate-buffered saline (PBS). Line 1–17 cells were seeded on 48-well culture plates at a density of 2.5 × 10^4^ cells/well and cultured for 24 h. Then, D-WMTA or Na-WMTA discs were gently placed on the cells (one disc per well). Culture plates with line 1–17 cells and WMTA discs were subsequently incubated for 7 or 28 days.

### Von Kossa staining

After 28 days of incubation, line 1–17 cells were fixed with 4% paraformaldehyde (Merck, Darmstadt, Germany) in DW. Cells were then washed with DW and mineralized nodule formation was confirmed by Von Kossa staining. Briefly, fixed cells were stained with 2% silver nitrate solution (Nacalai Tesque, Kyoto, Japan) in DW, in accordance with our previously described method^15^. Cells were then washed with running tap water and specimens were observed with an inverted microscope (BX41; Olympus, Tokyo, Japan).

### Quantitative reverse transcription polymerase chain reaction (qRT-PCR)

After 7 days of incubation, total RNA was isolated from line 1–17 cells using TRIzol Reagent (Invitrogen, Carlsbad, CA, USA). First‐strand complementary DNA was synthesized from 1 mg of total RNA using an ExScript RT reagent kit (Takara Bio Inc., Shiga, Japan). qRT‐PCR was performed with a KAPA Express Extract Kit (Kapa Biosystems, Wilmington, MA, USA) using a Thermal Cycler Dice Real Time System (Takara Bio Inc.). Specific primer sequences, annealing temperatures, and product sizes for each gene are listed in Supplementary Table 1. β‐Actin served as an internal control. Expression levels of the target genes were calculated using 2^−ΔΔCt^ method.

### Statistical analysis

All experiments were performed in triplicate or quadruplicate. All values are expressed as the mean ± standard deviation (SD). Statistical analysis was performed using one-way ANOVA, followed by the Bonferroni method for comparisons of three or more groups. P < 0.05 was considered statistically significant.

## Results

### Discoloration of WMTA discs immersed in sodium hypochlorite solution

**Figure 1:**
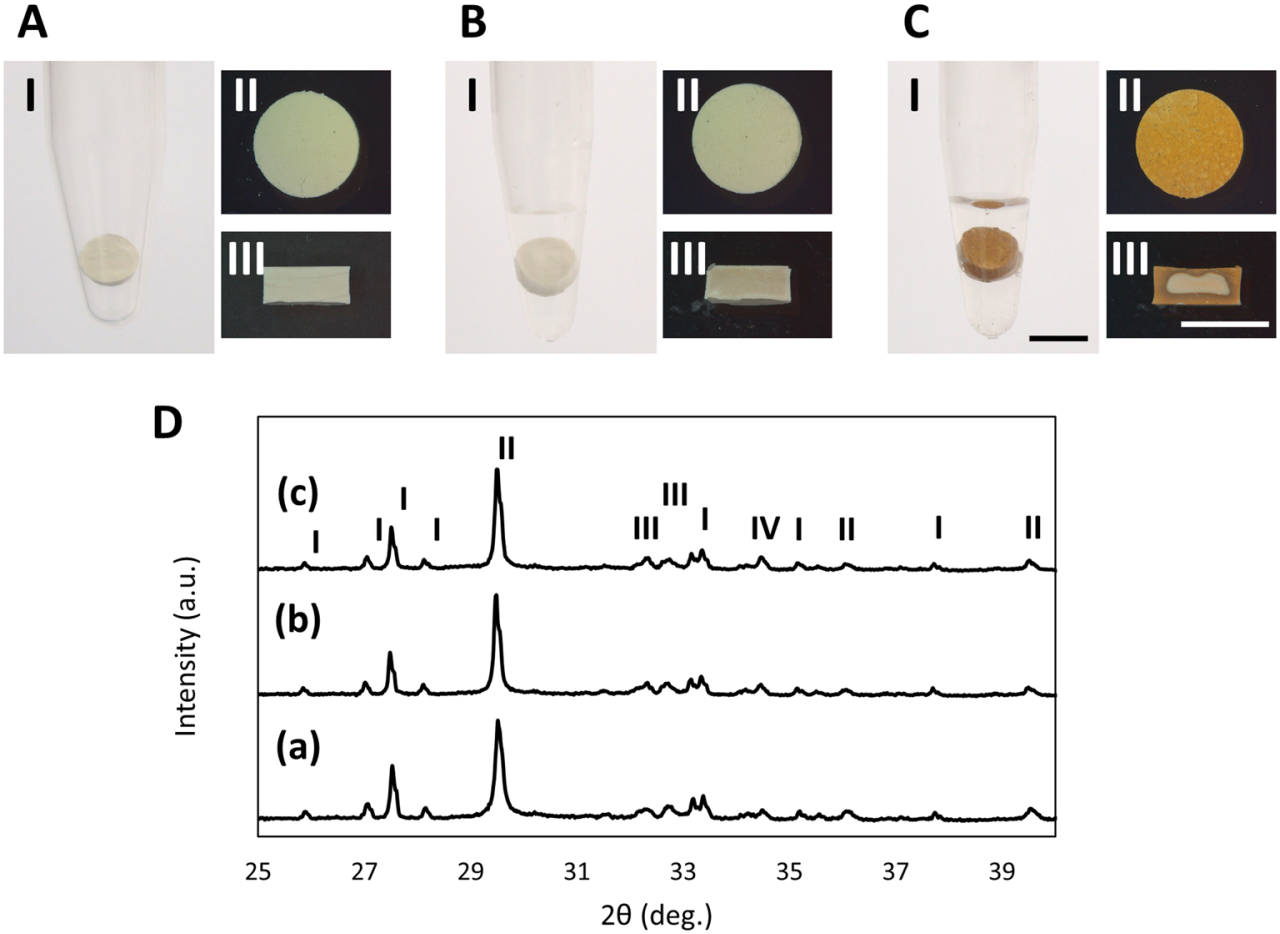
Photographic and x-ray diffraction analysis of WMTA discs immersed in NaOCl. (**A**) Images of WMTA discs before immersion (Cont-WMTA). (**B**,**C**) Images of WMTA discs after 24 h of immersion. WMTA discs were immersed in DW (D-WMTA) (**B**) or in NaOCl (Na-WMTA) (**C**). Photo (I) and stereomicroscopic (II) images of WMTA discs. (III) Stereomicroscopic images of cleaved WMTA discs. Bars = 5 mm. Experiments were performed in quadruplicate. (**D**) XRD patterns of Cont-WMTA (a), D-WMTA (b), and Na-WMTA (c). I: Bismite (Bi_2_O_3_, PDF#00-002-0498), II: Calcite (CaCO_3_, PDF#00-002-0629), III: Calcium silicate (Ca_2_SiO_4_, PDF#00-002-0843), IV: Calcium hydroxide (Ca[OH]_2_, PDF#01-070-5492).

Cont-WMTA exhibited yellowish white surface color (Fig. 1A[I, II]); the inside color was similar to the surface color (Fig. 1A[III]). D-WMTA exhibited a color nearly identical to Cont-WMTA both on the surface and inside (Fig. 1B[I–III]). However, Na-WMTA exhibited dark brown discoloration on the surface, which contrasted with Cont-WMTA and D-WMTA (Fig. 1C[I, II]). Na-WMTA also exhibited internal discoloration (Fig. 1C[III]). Notably, NaOCl induced discoloration of WMTA discs at concentrations from 2 to 0.01%; however, WMTA discs in contact with 0.001% and 0.0001% NaOCl exhibited a color similar to discs immersed in DW (Supplementary Fig. 1).

### Crystal structures of WMTA discs immersed in sodium hypochlorite solution

XRD analysis demonstrated that the main crystal structures of Cont-WMTA were bismite (Bi_2_O_3_: Fig. 1D[I]), calcite (CaCO_3_: Fig. 1D[II]), calcium silicate (Ca_2_SiO_4_: Fig. 1D[III]), and Ca (OH)_2_ (Fig. 1D[IV]). The main phases of D-WMTA and Na-WMTA were almost identical to the main phase of Cont-WMTA.

### Scanning electron microscopy and energy-dispersive X-ray analysis

**Figure 2:**
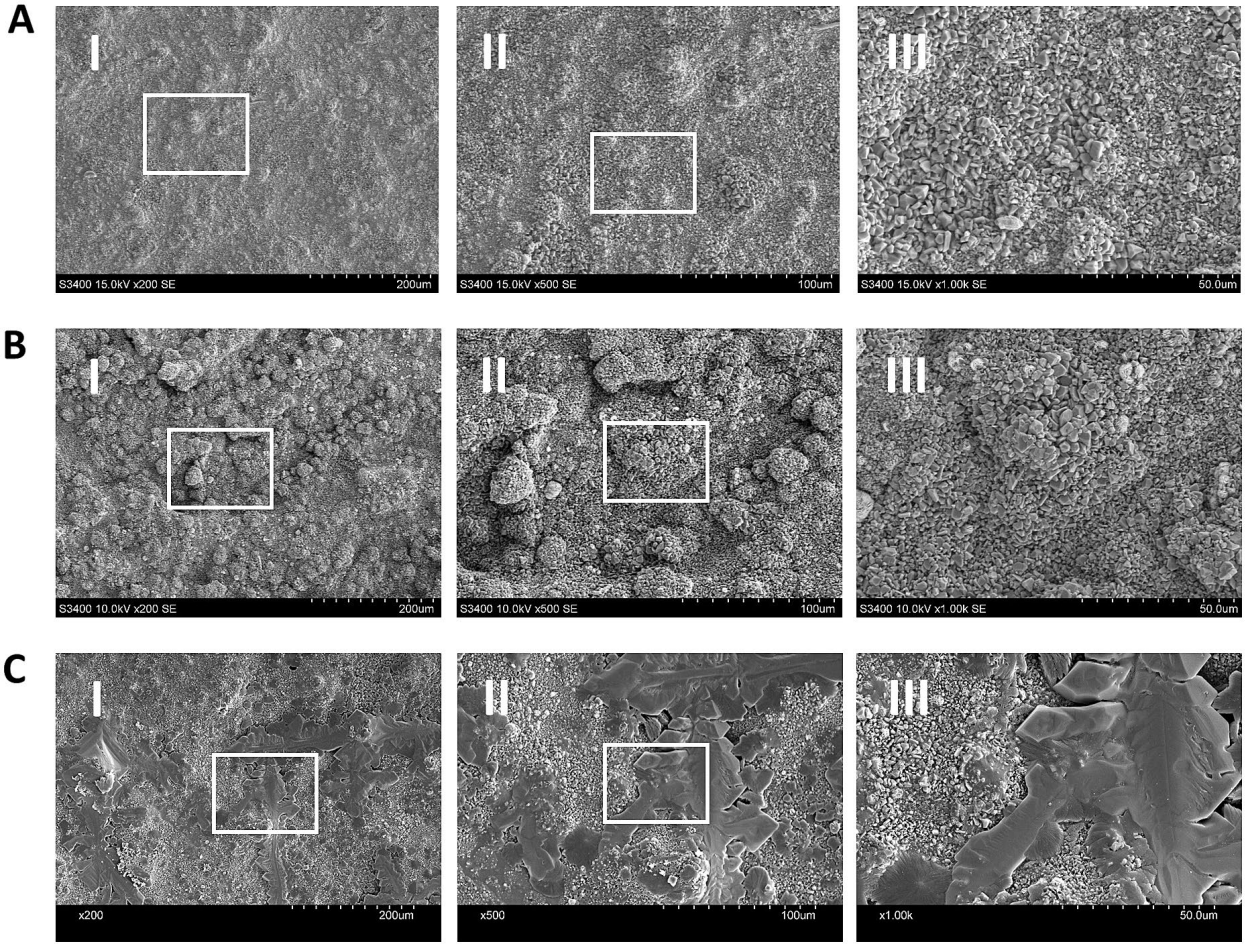
Scanning electron microscopy analysis of WMTA discs immersed in NaOCl. SEM micrographs obtained from Cont-WMTA (**A**), D-WMTA (**B**), and Na-WMTA (**C**). Original magnification: ×200 (I), ×500 (II), and ×1000 (III). White boxes in (I) and (II) indicate magnified areas.

SEM analysis revealed the formation of globular aggregate particles at 200× magnification on the surfaces of Cont-WMTA and D-WMTA (Fig. 2A[I],B[I]). Many polygonal crystals and small numbers of needle-like crystals were observed on these discs at 500× and 1000× magnification (Fig. 2A[II, III],B[II, III]). Globular aggregates consisting of polygonal and needle-like crystals were detected on the surfaces of Na-WMTA; however, many large and uneven structures were observed on the aggregates (Fig. 2C[I–III]). These structures were not observed on WMTA discs immersed in 0.1% (Supplementary Fig. 2A[I–III]), 0.5% (Supplementary Fig. 2B[I–III]), or 1% NaOCl (Supplementary Fig. 2C[I–III]). Notably, WMTA discs immersed in 2% NaOCl formed uneven structures; however, their numbers and sizes were smaller than the numbers and sizes on WMTA discs immersed in 5% NaOCl (Supplementary Fig. 2D[I–III]).

**Figure 3:**
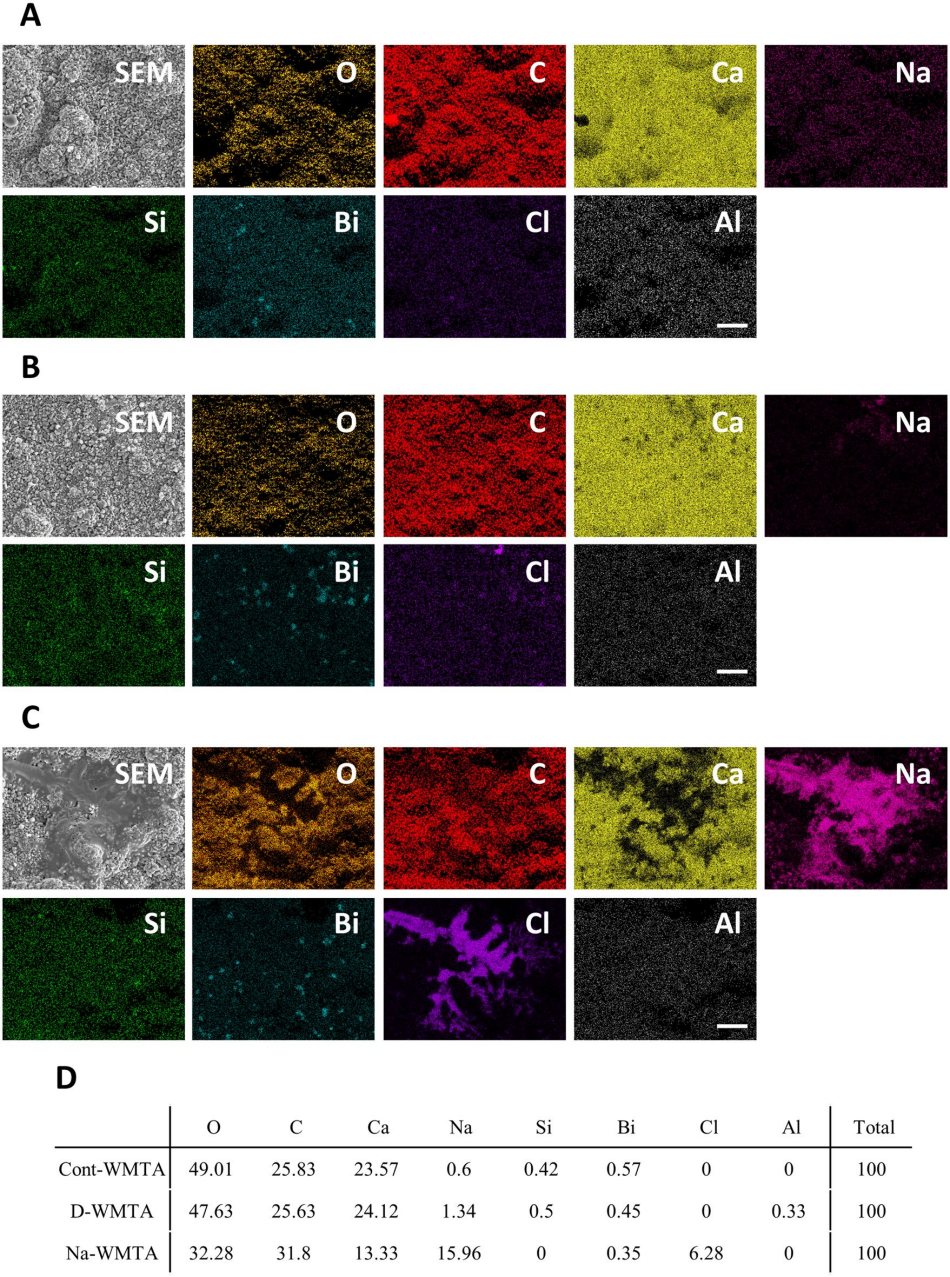
Elemental mapping by energy-dispersive X-ray analysis of WMTA discs immersed in NaOCl. EDX elemental mapping micrographs of Cont-WMTA (**A**), D-WMTA (**B**), and Na-WMTA (**C**). SEM, SEM micrographs; O (orange); C (red); Ca (yellow); Na (pink); Si, (green); Bi, (blue); Cl, (purple); Al, (white). Original magnification: ×1000. Bars = 20 µm. (**D**) Atomic percentage (At%) of Cont-WMTA, D-WMTA, and Na-WMTA.

EDX analysis demonstrated that the surfaces of Cont-WMTA and D-WMTA had similar elemental maps; they showed high peaks of O, C, and Ca; weak peaks of Na, Si, and Bi; and almost no peaks of Cl and Al (Fig. 3A,B,D, Supplementary Fig. 3A,B). However, the surfaces of Na-WMTA exhibited a distinct elemental map; Na and Cl were detected at sites from which Ca had disappeared (Fig. 3C, Supplementary Fig. 4A–D). Additionally, the ratio of Ca decreased and the ratios of Na and Cl increased in Na-WMTA, compared with those ratios in Cont-WMTA and D-WMTA (Fig. 3D, Supplementary Fig. 3C).

### Ca^2+^ release and pH in WMTA discs immersed in sodium hypochlorite solution

**Figure 4:**
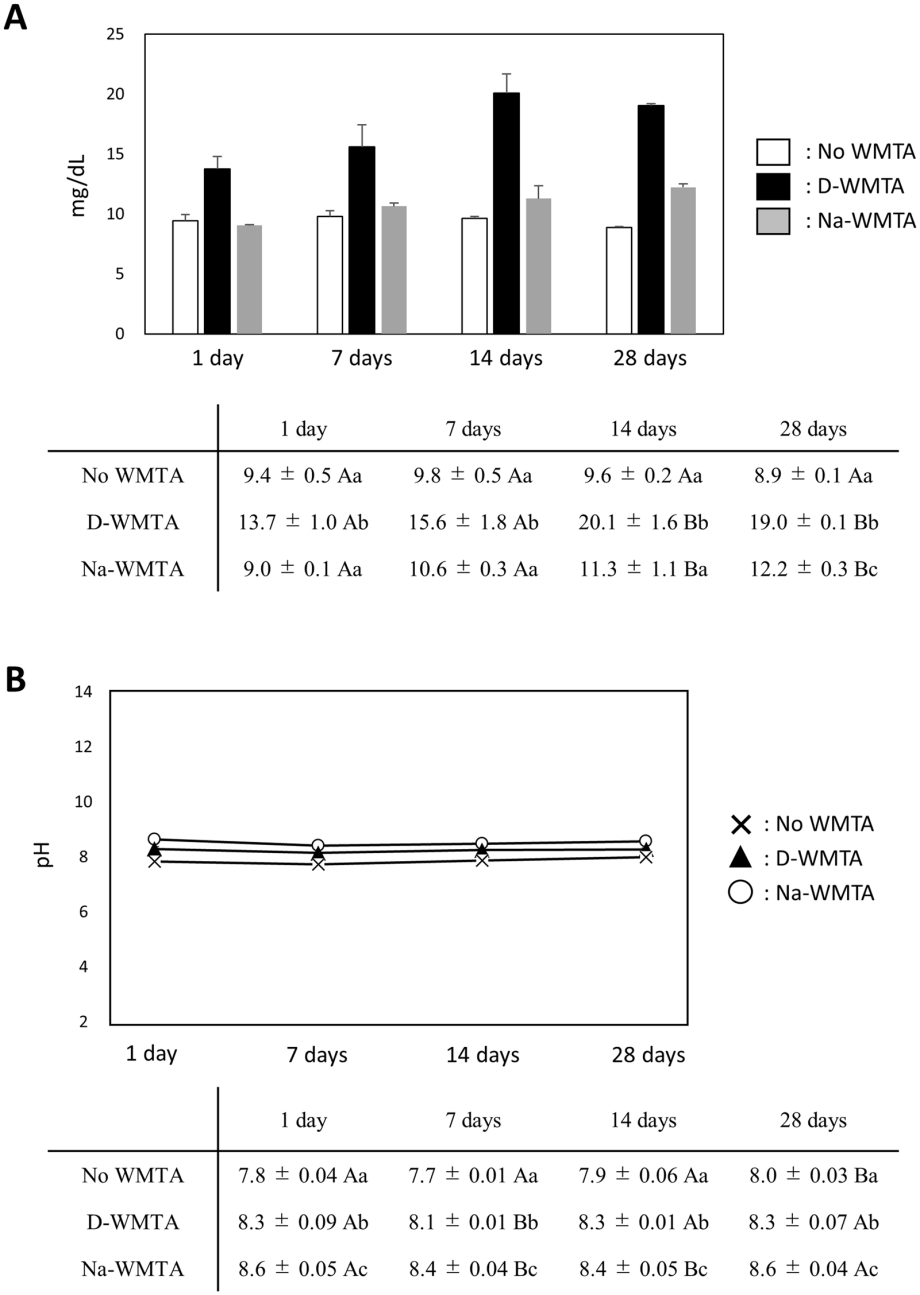
Effects of WMTA discs immersed in NaOCl on Ca^2+^ release and pH. Ca^2+^ amount (**A**) and pH (**B**) alteration in the medium. D-WMTA and Na-WMTA discs were immersed in CM for 1, 7, 14 and 28 days (n = 3 discs per time point). CM without WMTA discs (No WMTA) was used as a control. Different upper-case letters indicate statistically significant differences between WMTA discs. Different lower-case letters indicate statistically significant differences between time points. *P < 0.05.

The concentration of Ca^2+^ did not change in No WMTA over 28 days; however, CM with D-WMTA or Na-WMTA increased the concentration of Ca^2+^in a time-dependent manner (Fig. 4A). Cumulative Ca^2+^ in CM with D-WMTA was significantly higher than that in No WMTA at 1, 7, 14, and 28 days (Fig. 4A). However, Ca^2+^ release from Na-WMTA was significantly less than Ca^2+^ release from D-WMTA at 1, 7, 14, and 28 days (Fig. 4A). pH values showed minimal changes in No WMTA, CM with D-WMTA, and CM with Na-WMTA over 28 days (Fig. 4B).

### Effects of WMTA discs immersed in sodium hypochlorite solution on osteoblastic differentiation in a multipotent clonal human periodontal ligament cell line

**Figure 5:**
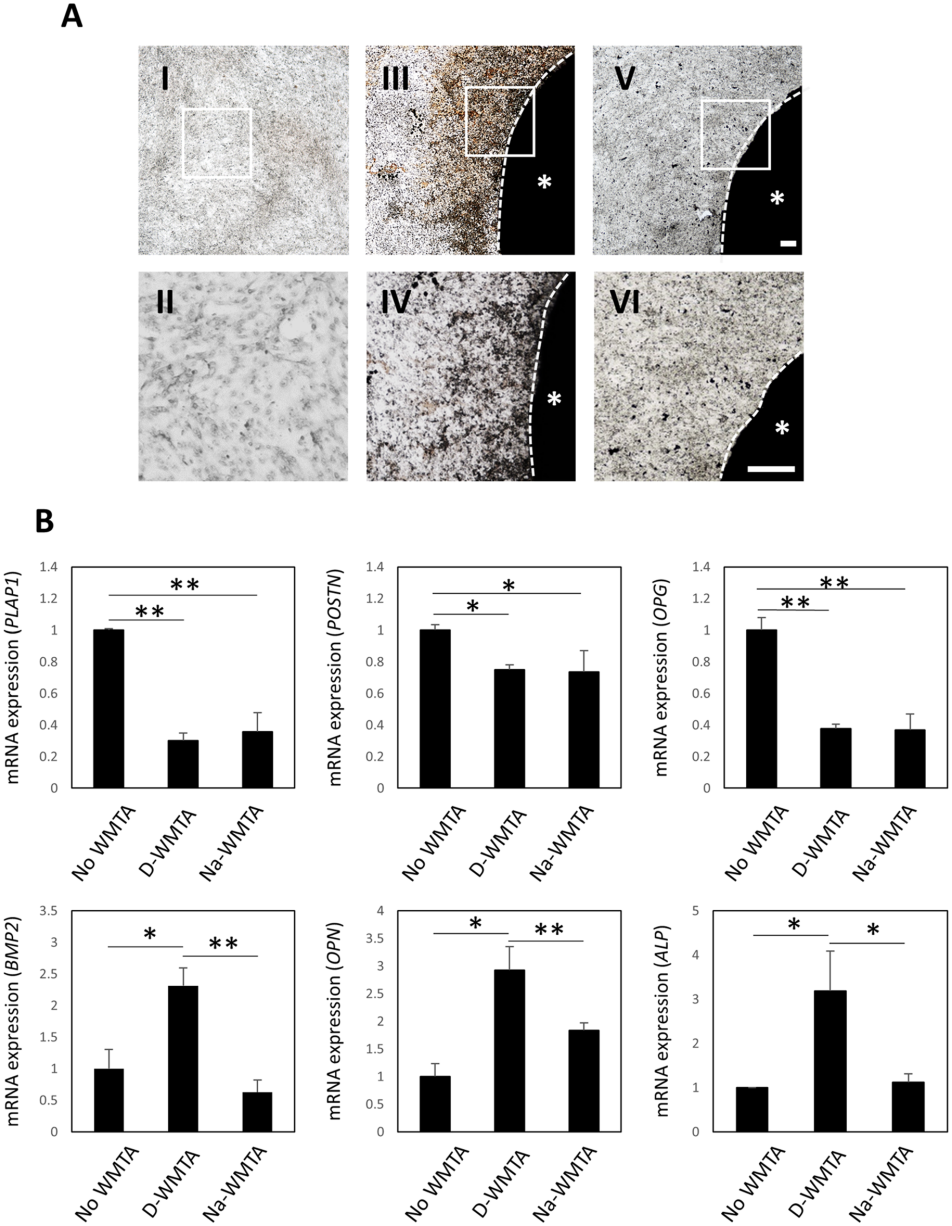
Effects of WMTA discs immersed in NaOCl on osteoblastic differentiation of line 1–17 cells. (**A**) Von Kossa staining images of line 1–17 cells. This line was cultured in No WMTA (I and II), CM with D-WMTA (III and IV), or CM with Na-WMTA (V and VI) for 28 days. (II, IV, and VI) show higher magnification images of the white boxes in (I, III, and V), respectively. Asterisks indicate WMTA discs and white dotted lines indicate their edges. Experiments were performed in quadruplicate. Representative data are shown. Bars = 100 µm. (**B**) Quantitative RT-PCR analysis of PDL-related genes (*PLAP1*, *POSTN*, and *OPG*) and osteoblast-related genes (*BMP2*, *OPN*, and *ALP*) in line 1–17 cells cultured in No WMTA, CM with D-WMTA, or CM with Na-WMTA. PLAP1, Periodontal ligament-associated protein 1; POSTN, Periostin; OPG, Osteoprotegerin; BMP2, Bone morphogenetic protein 2; OPN, Osteopontin; ALP, Alkaline phosphatase. These gene expression values were normalized to *Beta actin* (*β-act*) and No WMTA. *P < 0.05, **P < 0.01.

Line 1–17 cells cultured in No WMTA generated no Von Kossa staining-positive mineralized nodules (Fig. 5A[I, II]). This line cultured with D-WMTA formed extensive mineralized nodules around the discs (Fig. 5A[III, IV]). However, cells cultured with Na-WMTA produced few mineralized nodules (Fig. 5A[V, VI]). The mRNA expression levels of PDL-related genes (*PLAP1*, *POSTN*, and *OPG*) were significantly downregulated in line 1–17 cells cultured with D-WMTA and Na-WMTA, compared with cells cultured in No WMTA (Fig. 5B). Additionally, the mRNA expression levels of osteoblast-related genes (*BMP2*, *OPN*, and *ALP*) were significantly increased in line 1–17 cells cultured with D-WMTA, compared with cells cultured in No WMTA (Fig. 5B). The expression levels of these genes were significantly decreased in line 1–17 cells cultured with Na-WMTA, compared with cells cultured with D-WMTA (Fig. 5B).

## Discussion

This study was performed to investigate the structure of WMTA treated with NaOCl and determine the effects of NaOCl-exposed WMTA on osteoblastic differentiation in line 1–17 cells. NaOCl is the most widely accepted root canal irrigant^6^ and concentrations of NaOCl ranging from 0.5 to 6% are applied to root canals in clinical practice^16^. A previous report demonstrated that 3% and 5% NaOCl were more effective for the dissolution of pulp tissues, compared with 1% NaOCl^17^. Another study revealed that the activities of five strains of microorganisms were eliminated by 5.25% NaOCl; however, 2.5% NaOCl showed similar effects only on *Enterococcus faecalis*^18^. Additionally, a survey involving active members of the American Association of Endodontists demonstrated that more than 80% of respondents used concentrations of 5.25–8.25% NaOCl^19^. Based on those results, 5% NaOCl was used in this study.

Our study demonstrated that the color of set WMTA discs changed from yellowish-white to dark brown when the discs were immersed in 5% NaOCl for 24 h. This result was consistent with the findings in a previous report; Keskin et al. immersed set WMTA cylinders in 5% NaOCl for 24 h, which led to brown discoloration^20^. Moreover, Camilleri demonstrated that the exposure of 2% NaOCl for 24 h induced discoloration of WMTA cylinders^7^, while Voveraityte et al. reported that WMTA applied to the roots of human teeth irrigated with 2.5% NaOCl induced coronal discoloration of those teeth after 1 month^21^. These results suggested that NaOCl induced discoloration of WMTA, despite application at concentrations below 5%. Therefore, we also investigated whether concentrations of NaOCl below 2% induced discoloration of WMTA discs. Surprisingly, WMTA discs in contact with 0.01% NaOCl showed color alteration (Supplementary Fig. 1), suggesting that small amounts of NaOCl could induce discoloration of WMTA.

Notably, WMTA discs exhibited internal discoloration following NaOCl immersion, suggesting the penetration of NaOCl into WMTA discs. NaOCl has exhibited high penetration ability; penetration depths of 1% and 6% NaOCl into dentinal tubes were 77 and 123 µm after 2 min of treatment^22^. Nonetheless, some studies have demonstrated that WMTA has a high sealing ability^23–26^. These conflicting results might be because of differences in experimental design. Many previous reports used dye, bacterial, or endotoxin penetration techniques to evaluate WMTA sealing ability; however, the fluid filtration method using H_2_O revealed a high level of H_2_O filtration in WMTA^27^. Molecular size is generally important for compound permeability; the molecular size of H_2_O is considerably smaller than the molecular sizes of dye, bacteria, or endotoxin. Our results indicate that the molecular size of NaOCl is also smaller than the molecular sizes of dye, bacteria, or endotoxin; therefore, NaOCl could penetrate WMTA discs and induce internal discoloration.

WMTA sets through hydration of Ca_2_SiO_4_ to yield its hydrates and Ca(OH)_2_. In XRD analysis, all WMTA discs revealed a peak of Ca(OH)_2_ at 34.2° 2θ, which was consistent with a previous report^28^. However, Zapf et al. demonstrated that NaOCl suppressed the production of Ca(OH)_2_ in WMTA^8^. These different results might be explained by the timing of the NaOCl contact; set WMTA discs were immersed in NaOCl in this study and WMTA powder was mixed with NaOCl in the previous report. Additionally, Ca(OH)_2_ was identified in set WMTA, rather than in WMTA powder^28^. Based on these results, a large amount of Ca(OH)_2_ was presumably formed on the surfaces of set WMTA discs; therefore, NaOCl immersion might have partially eliminated Ca(OH)_2_ in the present study_._ To investigate this hypothesis, we examined the Ca distribution on the surfaces of Na-WMTA; we found that Na-WMTA exhibited a degree of Ca^2+^ release. Notably, our SEM and EDX examination demonstrated the formation of many large and uneven structures on the surfaces of Na-WMTA; Ca was not detected where these structures were observed. EDX analysis also revealed that Na and Cl were major elements of these structures. However, XRD examination revealed that the peak of NaCl was not present in Na-WMTA. Therefore, these structures were presumably amorphous structures mainly composed of Na and Cl, rather than crystals; furthermore, they were suspected to inhibit the formation of Ca(OH)_2_ on the surfaces of set WMTA discs.

A previous study suggested the importance of high pH and Ca^2+^ release for the induction of hard tissue formation in MTA^29^. Torabinejad et al. showed that the pH of DW containing set MTA rose to 12.5 over 24 h, and thereafter it remained constant^30^. However, the pH values of CM with D-WMTA and Na-WMTA reached approximately 8, which was almost identical to the pH of No WMTA and was lower than previously reported values. Generally, cell culture medium contains pH buffers such as Na_2_HPO_4_ and NaHCO_3_ to reduce pH-related damage in cells. Morita et al. showed that the pH of BHI broth medium containing set WMTA reached approximately 8^31^, which was consistent with our results.

After root perforation repairs, WMTA at the repaired sites is in contact with PDLSCs. The present study demonstrated the potential for NaOCl to penetrate into set WMTA. Therefore, we also investigated the effects of NaOCl-exposed WMTA on osteoblastic differentiation in line 1–17 cells. Line 1–17 cells cultured with D-WMTA and Na-WMTA formed mineralized particles around the discs; they also expressed lower levels of *PLAP1*, *POSTN*, and *OPG* genes, compared with cells cultured without WMTA discs. PLAP1 and POSTN are major extracellular proteins in PDL tissue^32^ and OPG is secreted from human PDL cells to regulate osteoclasts in PDL tissue^33^; therefore, these proteins (and the genes that encode them) are regarded as PDL-related markers. A previous report revealed that human PDLSCs expressed high levels of *PLAP1* and *POSTN*; however, the expression levels of these genes were extremely low in human osteoblasts^34^, suggesting that line 1–17 cells exposed to D-WMTA and Na-WMTA differentiated into osteoblast-like cells. Furthermore, line 1–17 cells cultured with Na-WMTA expressed lower levels of *BMP2*, *OPN*, and *ALP* genes, compared with cells cultured with D-WMTA. OPN and ALP are well-known osteoblast markers and their expression levels were increased in dog PDL tissue in contact with WMTA^35^. BMP2 is reportedly involved in osteoblast maturation and plays important roles in promoting osteoblastic differentiation in human PDL cells^36^. Our previous report revealed the upregulation of *BMP2* in human PDL cells that had been treated with set WMTA^37^. Based on these results, Na-WMTA could stimulate hard tissue formation; however, its stimulating effect would be less robust than the effect of D-WMTA.

In summary, we have shown that NaOCl penetrates set WMTA discs and induces both surface and internal discoloration of those discs. NaOCl did not alter the crystal structures of the discs, although it induced the formation of many large and uneven structures on the disc surfaces. These structures were mainly composed of Na and Cl; they reduced Ca^2+^ release from WMTA discs. NaOCl inhibited WMTA-induced mineralized nodule formation and osteoblast-related gene expression in line 1–17 cells. Therefore, NaOCl should be firmly removed from root canals that are repaired by WMTA after root canal treatment to avoid its discoloration effects while maintaining its ability to stimulate hard tissue formation.

## Supplementary Information

Supplementary Information.
