# Supplementary material for: Mineral trioxide aggregate immersed in sodium hypochlorite reduce the osteoblastic differentiation of human periodontal ligament stem cells
Source: Sci Rep. 2021 Nov 11;11:22091. doi: 10.1038/s41598-021-01545-3 (PMC8585953; doi:10.1038/s41598-021-01545-3)
Supplement: Supplementary file 1 — Supplementary Information. [file 41598_2021_1545_MOESM1_ESM.pdf]

Supplementary Information for

Mineral trioxide aggregate immersed in sodium hypochlorite reduce the osteoblastic differentiation of human periodontal ligament stem cells

Kozue Yamashita[1)], Atsushi Tomokiyo[2)*], Taiga Ono[2)], Keita Ipposhi[1)], M. Anas Alhasan[1)], Akira Tsuchiya[4)], Sayuri Hamano[1,3)], Hideki Sugii[1)], Shinichiro Yoshida[2)], Tomohiro Itoyama[2)], Hidefumi Maeda[1,2)]

1) Department of Endodontology and Operative Dentistry, Faculty of Dental Science, Kyushu University
2) Department of Endodontology, Kyushu University Hospital
3) OBT Research Center Faculty of Dental Science, Kyushu University
4) Department of Biomaterials, Faculty of Dental Science, Kyushu University

1~4) 3-1-1 Maidashi, Higashi-ku, Fukuoka-shi, Fukuoka, 812-8582, Japan

***Corresponding author**
Name: Atsushi Tomokiyo
Institution: Department of Endodontology, Kyushu University Hospital
Address: 3-1-1, Maidashi, Higashi-ku, Fukuoka-shi, Fukuoka, 812-8582, JAPAN
Phone Number: +81926426432
E-mail: tomokiyo@dent.kyushu-u.ac.jp

Target gene	Gene bank ID	Primer sequence forward/reverse	Product size (bp)	Annealing temperature (°C)
PLAP1	NM_017680.5	5'-ATGGGAGTCTTGCTAACATACCAC-3'/ 5'-CAGAAGTCATTTACTCCCACTCTTG-3'	154	60
POSTN	NM_006475.2	5'-CATTGATGGAGTGCCTGTGGA-3'/ 5'-CAATGAATTTGGTGACCTTGGTG-3'	167	60
OPG	NM_002546.4	5'-CTCGAAGGTGAGGTTAGCATGTC-3'/ 5'-TGGCACCAAAGTAAACGCAGAG-3'	196	60
BMP2	NM_001200.4	5'-TCCACTAATCATGCCATTGTTCAGA-3'/ 5'-GGGACACAGCATGCCTTAGGA-3'	74	60
OPN	NM_001040058.2	5'-ACACATATGATGGCCGAGGTGA-3'/ 5'-TGTGAGGTGATGTCCTCGTCTGT-3'	40	60
ALP	NM_000478.6	5'-GGACCATTCCCACGTCTTCAC-3'/ 5'-CCTTGTAGCCAGGCCCATTG-3'	137	60
β-act	NM_001101.5	5'-ATTGCCGACAGGATGCAGA-3'/ 5'-GAGTACTTGCGCTCAGGAGGA-3'	89	60

Supplementary Table 1. GenBank ID, primer sequences, product sizes, and annealing temperatures for quantitative reverse transcription polymerase chain reaction

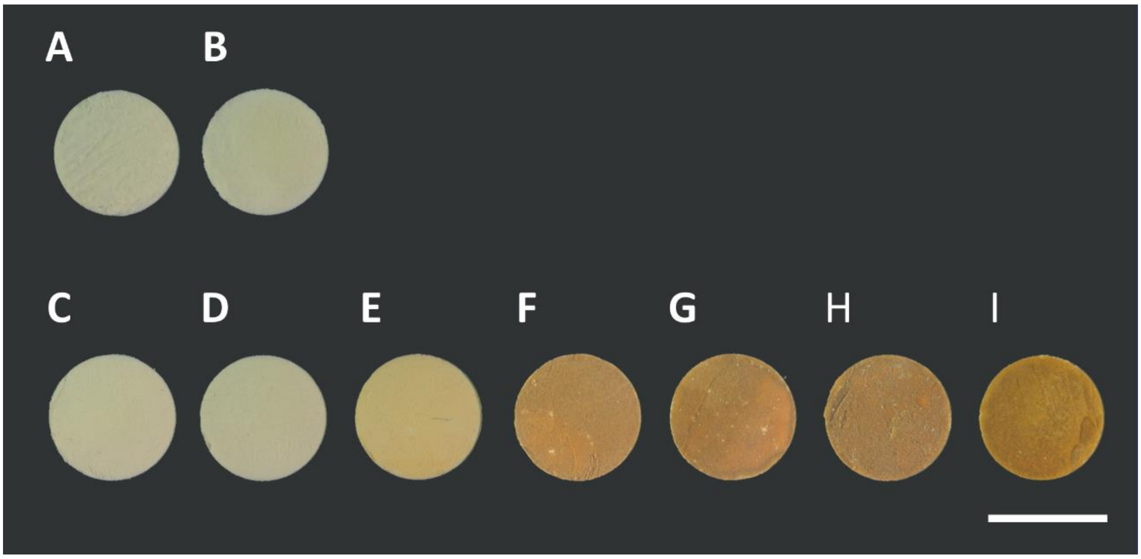

Supplementary Figure 1. Photographic analysis of WMTA discs immersed in various concentrations of NaOCl

Stereomicroscopic images of WMTA discs before (A) or after 24 hours of immersion (B–I). WMTA discs were immersed in DW (B) or in 0.0001% (C), 0.001% (D), 0.01% (E), 0.1% (F), 0.5% (G), 1% (H), or 2% (I) NaOCl. Bar = 5 mm.

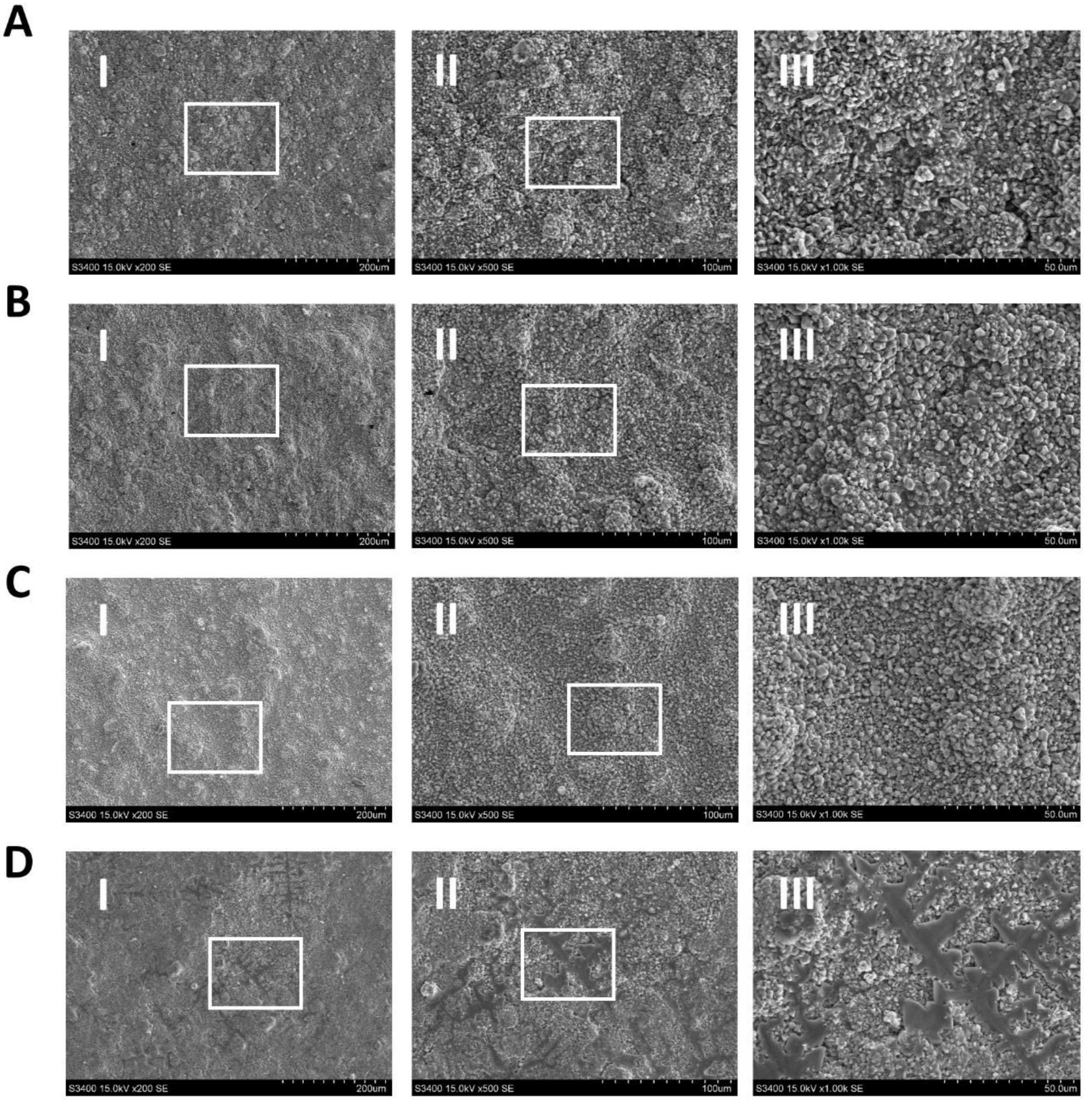

Supplementary Figure 2. Scanning electron microscopy analysis of WMTA discs immersed in various concentrations of NaOCl

SEM micrographs obtained from WMTA discs immersed in 0.1% (A), 0.5% (B), 1% (C), or 2% (D) NaOCl. Original magnification: 200× (I), 500× (II), and 1000× (III). White boxes in (I) and (II) indicate magnified areas.

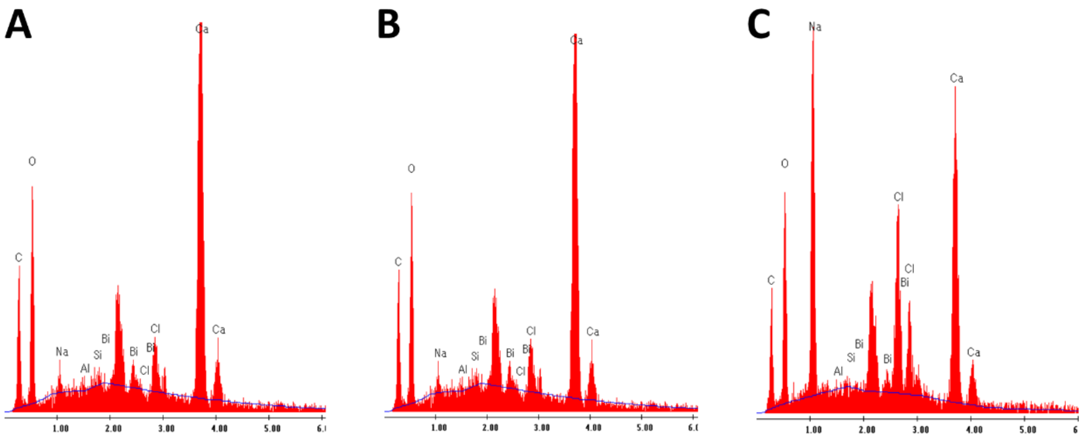

Supplementary Figure 3. Energy-dispersive X-ray spectroscopy of WMTA discs immersed in NaOCl

EDX spectroscopy containing O, C, Ca, Na, Si, Bi, Cl, and Al in Cont-WMTA (A), D-WMTA (B), and Na-WMTA (C).

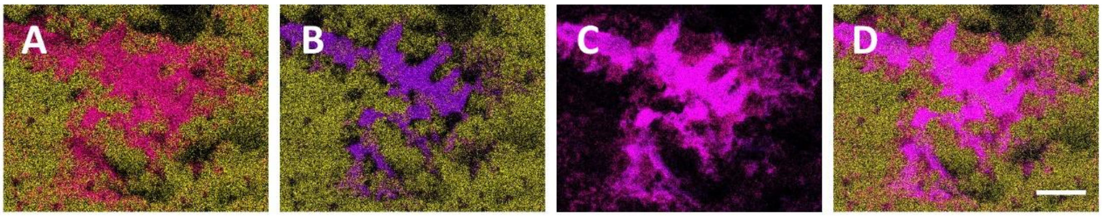

Supplementary Figure 4. Merge images of elemental mapping in WMTA discs immersed in NaOCl

Merged elemental mapping images of Ca and Na (A), Ca and Cl (B), Na and Cl (C), and Ca, Na, and Cl (D) in Na-WMTA. Yellow, pink, and purple colors indicate Ca, Na, and Cl, respectively. Original magnification: 1000×. Bars = 20 μm.
